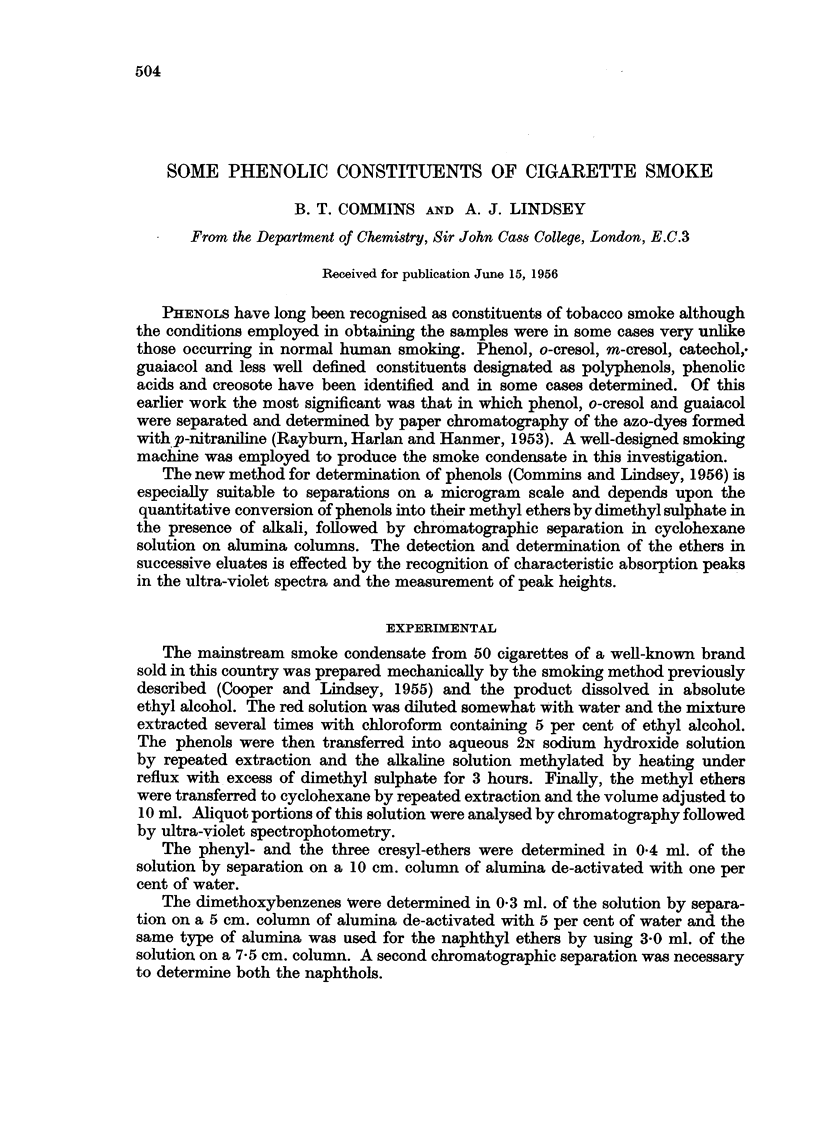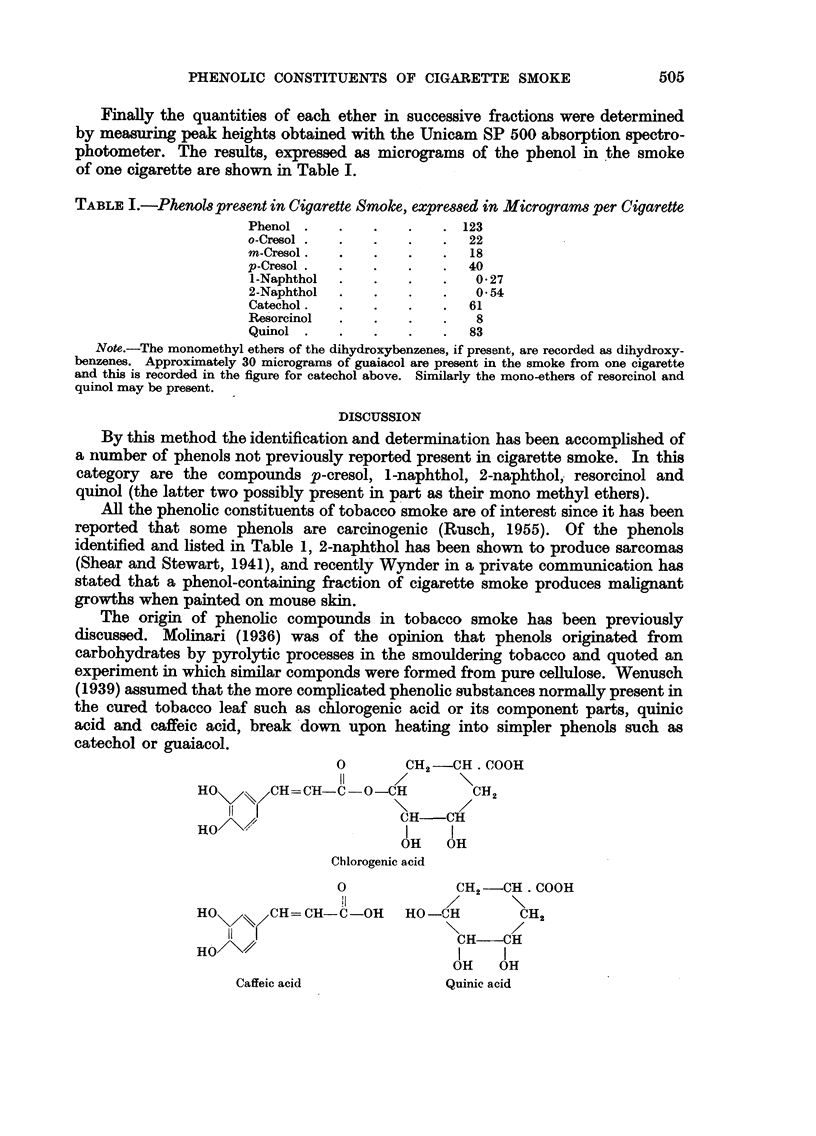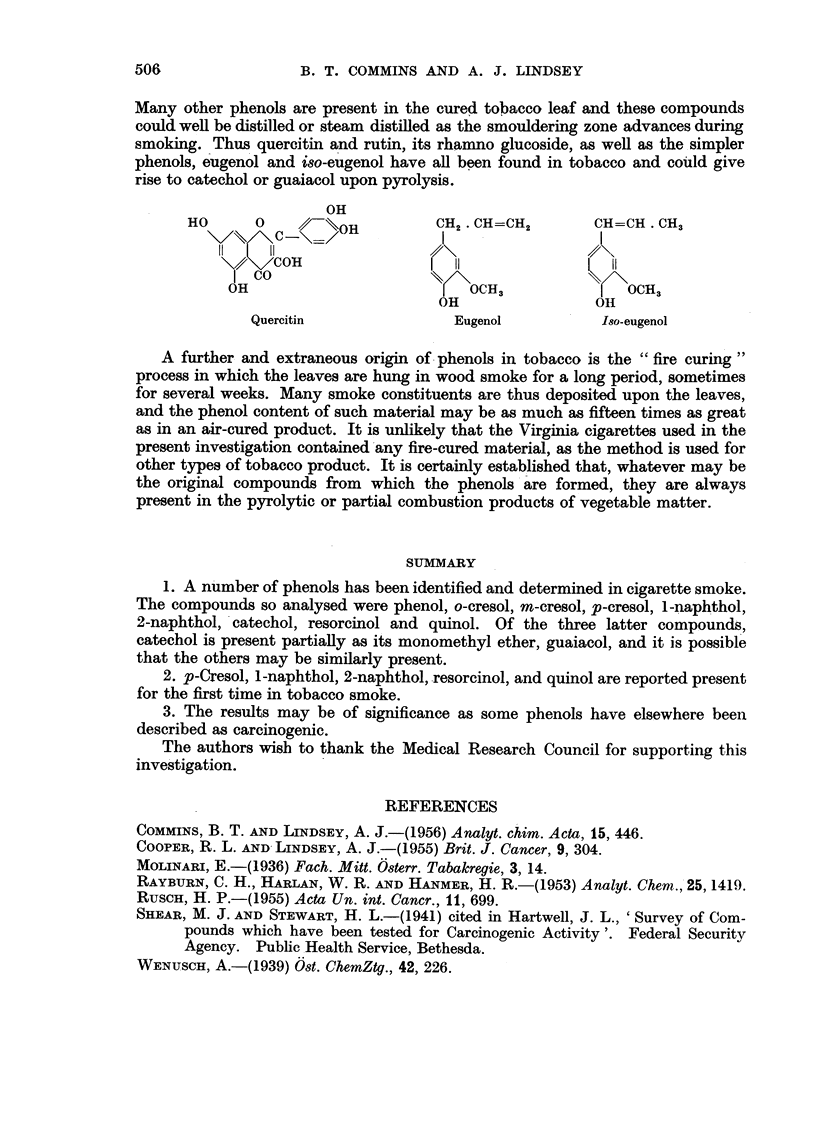# Some Phenolic Constituents of Cigarette Smoke

**DOI:** 10.1038/bjc.1956.57

**Published:** 1956-09

**Authors:** B. T. Commins, A. J. Lindsey


### SOME PHENOLIC CONSTITUENTS OF CIGARETTE SMOKE

B. T. COMMINS AND A. J. LINDSEY

*From the Department of Chemistry, Sir John Cass College, London, E.C.3*

Received for publication June 15, 1956

PHENOLS have long been recognised as constituents of tobacco smoke although the conditions employed in obtaining the samples were in some cases very unlike those occurring in normal human smoking. Phenol, *o*-cresol, *m*-cresol, catechol, guaiacol and less well defined constituents designated as polyphenols, phenolic acids and creosote have been identified and in some cases determined. Of this earlier work the most significant was that in which phenol, *o*-cresol and guaiacol were separated and determined by paper chromatography of the azo-dyes formed with *p*-nitraniline (Rayburn, Harlan and Hanmer, 1953). A well-designed smoking machine was employed to produce the smoke condensate in this investigation.

The new method for determination of phenols (Commins and Lindsey, 1956) is especially suitable to separations on a microgram scale and depends upon the quantitative conversion of phenols into their methyl ethers by dimethyl sulphate in the presence of alkali, followed by chromatographic separation in cyclohexane solution on alumina columns. The detection and determination of the ethers in successive eluates is effected by the recognition of characteristic absorption peaks in the ultra-violet spectra and the measurement of peak heights.

### EXPERIMENTAL

The mainstream smoke condensate from 50 cigarettes of a well-known brand sold in this country was prepared mechanically by the smoking method previously described (Cooper and Lindsey, 1955) and the product dissolved in absolute ethyl alcohol. The red solution was diluted somewhat with water and the mixture extracted several times with chloroform containing 5 per cent of ethyl alcohol. The phenols were then transferred into aqueous 2N sodium hydroxide solution by repeated extraction and the alkaline solution methylated by heating under reflux with excess of dimethyl sulphate for 3 hours. Finally, the methyl ethers were transferred to cyclohexane by repeated extraction and the volume adjusted to 10 ml. Aliquot portions of this solution were analysed by chromatography followed by ultra-violet spectrophotometry.

The phenyl- and the three cresyl-ethers were determined in 0·4 ml. of the solution by separation on a 10 cm. column of alumina de-activated with one per cent of water.

The dimethoxybenzenes were determined in 0·3 ml. of the solution by separation on a 5 cm. column of alumina de-activated with 5 per cent of water and the same type of alumina was used for the naphthyl ethers by using 3·0 ml. of the solution on a 7·5 cm. column. A second chromatographic separation was necessary to determine both the naphthols.

Finally the quantities of each ether in successive fractions were determined by measuring peak heights obtained with the Unicam SP 500 absorption spectrophotometer. The results, expressed as micrograms of the phenol in the smoke of one cigarette are shown in Table I.

TABLE I.—*Phenols present in Cigarette Smoke, expressed in Micrograms per Cigarette*

| | |
|---|---|
| Phenol | 123 |
| *o*-Cresol | 22 |
| *m*-Cresol | 18 |
| *p*-Cresol | 40 |
| 1-Naphthol | 0·27 |
| 2-Naphthol | 0·54 |
| Catechol | 61 |
| Resorcinol | 8 |
| Quinol | 83 |

*Note.*—The monomethyl ethers of the dihydroxybenzenes, if present, are recorded as dihydroxybenzenes. Approximately 30 micrograms of guaiacol are present in the smoke from one cigarette and this is recorded in the figure for catechol above. Similarly the mono-ethers of resorcinol and quinol may be present.

### DISCUSSION

By this method the identification and determination has been accomplished of a number of phenols not previously reported present in cigarette smoke. In this category are the compounds *p*-cresol, 1-naphthol, 2-naphthol, resorcinol and quinol (the latter two possibly present in part as their mono methyl ethers).

All the phenolic constituents of tobacco smoke are of interest since it has been reported that some phenols are carcinogenic (Rusch, 1955). Of the phenols identified and listed in Table 1, 2-naphthol has been shown to produce sarcomas (Shear and Stewart, 1941), and recently Wynder in a private communication has stated that a phenol-containing fraction of cigarette smoke produces malignant growths when painted on mouse skin.

The origin of phenolic compounds in tobacco smoke has been previously discussed. Molinari (1936) was of the opinion that phenols originated from carbohydrates by pyrolytic processes in the smouldering tobacco and quoted an experiment in which similar componds were formed from pure cellulose. Wenusch (1939) assumed that the more complicated phenolic substances normally present in the cured tobacco leaf such as chlorogenic acid or its component parts, quinic acid and caffeic acid, break down upon heating into simpler phenols such as catechol or guaiacol.

Chlorogenic acid

Caffeic acid

Quinic acid

Many other phenols are present in the cured tobacco leaf and these compounds could well be distilled or steam distilled as the smouldering zone advances during smoking. Thus quercitin and rutin, its rhamno glucoside, as well as the simpler phenols, eugenol and *iso*-eugenol have all been found in tobacco and could give rise to catechol or guaiacol upon pyrolysis.

Quercitin

Eugenol

*Iso*-eugenol

A further and extraneous origin of phenols in tobacco is the " fire curing " process in which the leaves are hung in wood smoke for a long period, sometimes for several weeks. Many smoke constituents are thus deposited upon the leaves, and the phenol content of such material may be as much as fifteen times as great as in an air-cured product. It is unlikely that the Virginia cigarettes used in the present investigation contained any fire-cured material, as the method is used for other types of tobacco product. It is certainly established that, whatever may be the original compounds from which the phenols are formed, they are always present in the pyrolytic or partial combustion products of vegetable matter.

### SUMMARY

1. A number of phenols has been identified and determined in cigarette smoke. The compounds so analysed were phenol, *o*-cresol, *m*-cresol, *p*-cresol, 1-naphthol, 2-naphthol, catechol, resorcinol and quinol. Of the three latter compounds, catechol is present partially as its monomethyl ether, guaiacol, and it is possible that the others may be similarly present.

2. *p*-Cresol, 1-naphthol, 2-naphthol, resorcinol, and quinol are reported present for the first time in tobacco smoke.

3. The results may be of significance as some phenols have elsewhere been described as carcinogenic.

The authors wish to thank the Medical Research Council for supporting this investigation.

### REFERENCES

Commins, B. T. and Lindsey, A. J.—(1956) *Analyt. chim. Acta*, **15**, 446.
Cooper, R. L. and Lindsey, A. J.—(1955) *Brit. J. Cancer*, **9**, 304.
Molinari, E.—(1936) *Fach. Mitt. Österr. Tabakregie*, **3**, 14.
Rayburn, C. H., Harlan, W. R. and Hanmer, H. R.—(1953) *Analyt. Chem.*, **25**, 1419.
Rusch, H. P.—(1955) *Acta Un. int. Cancr.*, **11**, 699.
Shear, M. J. and Stewart, H. L.—(1941) cited in Hartwell, J. L., ' Survey of Compounds which have been tested for Carcinogenic Activity '. Federal Security Agency. Public Health Service, Bethesda.
Wenusch, A.—(1939) *Öst. ChemZtg.*, **42**, 226.